# Estimation of Genomic Breed Composition for Purebred and Crossbred Animals Using Sparsely Regularized Admixture Models

**DOI:** 10.3389/fgene.2020.00576

**Published:** 2020-06-11

**Authors:** Yangfan Wang, Xiao-Lin Wu, Zhi Li, Zhenmin Bao, Richard G. Tait, Stewart Bauck, Guilherme J. M. Rosa

**Affiliations:** ^1^Ministry of Education Key Laboratory of Marine Genetics and Breeding, College of Marine Life Science, Ocean University of China, Qingdao, China; ^2^Department of Animal Sciences, University of Wisconsin, Madison, WI, United States; ^3^Biostatistics and Bioinformatics, Neogen GeneSeek, Lincoln, NE, United States; ^4^Department of Animal Science, University of Wyoming, Laramie, WY, United States; ^5^Laboratory for Marine Fisheries Science and Food Production Processes, Qingdao National Laboratory for Marine Science and Technology, Qingdao, China

**Keywords:** admixture models, breed composition, bovine, linear regression, SNP, sparse regularization, nonconvex penalty

## Abstract

A variety of statistical methods, such as admixture models, have been used to estimate genomic breed composition (GBC). These methods, however, tend to produce non-zero components to reference breeds that shared some genomic similarity with a test animal. These non-essential GBC components, in turn, offset the estimated GBC for the breed to which it belongs. As a result, not all purebred animals have 100% GBC of their respective breeds, which statistically indicates an elevated false-negative rate in the identification of purebred animals with 100% GBC as the cutoff. Otherwise, a lower cutoff of estimated GBC will have to be used, which is arbitrary, and the results are less interpretable. In the present study, three admixture models with regularization were proposed, which produced sparse solutions through suppressing the noise in the estimated GBC due to genomic similarities. The regularization or penalty forms included the L1 norm penalty, minimax concave penalty (MCP), and smooth clipped absolute deviation (SCAD). The performances of these regularized admixture models on the estimation of GBC were examined in purebred and composite animals, respectively, and compared to that of the non-regularized admixture model as the baseline model. The results showed that, given optimal values for λ, the three sparsely regularized admixture models had higher power and thus reduced the false-negative rate for the breed identification of purebred animals than the non-regularized admixture model. Of the three regularized admixture models, the two with a non-convex penalty outperformed the one with L1 norm penalty. In the Brangus, a composite cattle breed, estimated GBC were roughly comparable among the four admixture models, but all the four models underestimated the GBC for these composite animals when non-ancestral breeds were included as the reference. In conclusion, the admixture models with sparse regularization gave more parsimonious, consistent and interpretable results of estimated GBC for purebred animals than the non-regularized admixture model. Nevertheless, the utility of regularized admixture models for estimating GBC in crossbred or composite animals needs to be taken with caution.

## Introduction

The estimation of genomic breed composition (GBC) of individual animals is useful in many aspects, such as predicting heterosis (Akanno et al., 2018), correcting population stratification effects in genetic association studies (Jiang et al., 2010; Mebratie et al., 2019), understanding the population structure and breeding history of the breeds of interest (Gobena et al., 2018), and making management decisions for crossbreeding programs (Pickrell and Pritchard, 2012; Akanno et al., 2018). In the past decades, pedigree information has been used to determine the breed composition of animals (Frkonja et al., 2012). The reliability of pedigree-estimated breed composition, however, can be compromised by missing, inaccurate, or incomplete records (vanRaden and Cooper, 2015). Another advantage with a pedigree-based estimator is that it yields the same GBC estimates for full-sib progenies of the same family. In reality, they can vary drastically in their actual genomic composition inherited from ancestors as the result of crossing-overs and chromosomal assortments taking place during meiosis. Instead, GBC can be estimated more accurately using genomic data, such as SNPs (Chiang et al., 2010; Kuehn et al., 2011; He et al., 2018) and sequence data (Bansal and Libiger, 2015; Taliun et al., 2017).

A variety of statistical methods and software packages have been developed to estimate GBC (Alexander et al., 2009; Kuehn et al., 2011; Frkonja et al., 2012; Bansal and Libiger, 2015). For example, a likelihood-based admixture model (vanRaden and Cooper, 2015; He et al., 2018) has been widely used. It postulates that a genotype of an SNP for a given animal is a random event following a probability being a mixture of the corresponding allele frequencies of its ancestors or ancestral breeds (Bansal and Libiger, 2015). A challenge with this model is that it tends to produce non-zero GBC components produced to reference breeds that shared genomic similarities with a test animal, which in turn offsets the estimated GBC for the breed to which this animal belongs. The consequence is that not all purebred animals have 100% estimated GBC of their respective breeds, which we refer to as the “Impure purebred Paradox.” Statistically, it indicates an elevated false-negative rate in the identification of purebred animals. The same situation happens with other statistical models such as linear regression. In dairy cattle, for example, the Council of Dairy Cattle Breeding (CDCB) in the USA has established a procedure termed Breed Base Representation (BBR) representing five dairy purebred reference groups (PRG): Ayrshire, Brown Swiss, Guernsey, Holstein, and Jersey. The measure of the same name estimated the genomic breed composition of individual animals using linear regression, with the estimates restricted to be between 0 and 100% for each PRG and summed up to 1 per genotyped animal. Their results showed that the mean BBR percentages were 94.8, 97.0, 97.8, 99.0, and 96.5%, respectively for all males genotyped for these breeds (201,283 animals), and 95.0, 97.1, 96.9, 98.9, and 96.5%, respectively, for all genotyped females (994,949 animals). Similar results were reported in beef cattle as well by Kuehn et al. (2011), who estimated GBC in seven breeds using linear regression. Their results showed that the regression coefficients varied from 0.737 (Angus) to 0.981 (Hereford). The regression coefficients were low for Angus (0.737) and Red Angus (0.883) because these two beef breeds share a high genetic similarity.

In the present study, regularized admixture methods were utilized to produce sparse solutions of admixture coefficients, thus imposing penalties on small, non-essential components due to genomic similarity. Three forms of sparse regularization were incorporated into the admixture models, which included the L1 norm penalty, minimax concave (MCP) penalty, and smooth clipped absolute deviation (SCAD). The L1 norm is the most commonly used convex surrogate (Tibshirani, 1996), whereas the other two are non-convex (Fan and Li, 2001; Zhang, 2010). The difference between convex optimization and non-convex optimization is that the former has one minimum, and hence the local optimum is also the global optimum. However, the latter can have multiple local minima, which are not all the same as the global minimum (Zhao et al., 2018). Nonconvex penalties can often lead to a better recovery in signals or variable selection in machine learning but at the expense of introducing a more challenging optimization problem (Jiao et al., 2016). The purpose of the present study was to evaluate the performance of the three sparsely regularized admixture models in the estimation of GBC for purebred and composite animals, respectively, in comparison with the non-regularized admixture model as the baseline model (Bansal and Libiger, 2015).

## Materials and Methods

### Animals and Genotype Data

The dataset used in the present study included 107,593 animals from ten breeds, nine pure breeds, and one composite breed. All these animals were genotyped on the GeneSeek Genomic Profiler (GGP) bovine 50 K version 1 SNP chip (49,463 SNPs), except that 349 Brahman animals were genotyped on the Illumina 777K bovine SNP chip (777,962 SNPs). The reference populations consisted of eight *Bos taurus taurus* breeds and one Bos *taurus indicus* cattle breed. The former included two dairy breeds (Holstein and Jersey) and six beef breeds (Angus, Hereford, Limousine, Shorthorn, Simmental, and Wagyu). Brahman is the only *indicus* cattle breed used in the present study. Summary statistics of the reference animals and their genotypes were shown in Table 1.

**Table 1:** Descriptive statistics of genotype data for the ten cattle breeds used in the present study.

**Breed**	**Number of genotyped animals^a^**	**Number of SNPs**	**Mean FreqA (SD)^b^**
Angus	20,359 (20,322)	49,463	0.492 (0.247)
Brahman	349 (349)	777,962	0.439 (0.343)
	68 (43)	49,463	0.431 (0.363)
Brangus	3,605	49,463	0.477 (0.231)
Hereford	2,423 (2,421)	49,463	0.496 (0.271)
Holstein	20,350 (20,246)	49,463	0.489 (0.254)
Jersey	15,689 (15,607)	49,463	0.489 (0.288)
Limousine	5,043 (5,041)	49,463	0.490 (0.228)
Shorthorn	1,232 (1,218)	49,463	0.491 (0.258)
Simmental	14,754 (14,727)	49,463	0.490 (0.226)
Wagyu	23,721 (21,844)	49,463	0.483 (0.302)

a *In the brackets are the number of genotyped animals remained after excluding outliers*.

b *Mean FreqA (SD) = mean (standard deviation) of allele A frequencies of genotyped SNP for each breed*.

Genomic breed composition was estimated based on SNP panels. The largest panel had 15,708 SNPs (referred to as the 16K SNP panel) which were common SNPs across five commercial bovine SNP chips, namely, Illumina Bovine high-density (HD or 777K) chip, GGP ultra-high-density (UHD or 150K) SNP chip, GGP HD (80K) SNP chip, GGP 50K version 1 SNP chip, and GGP low-density (LD or 40K) version 4 SNP chip. The main reason for us to use the shared content of these commercial SNP chips was to facilitate the estimation of GBC using currently available SNP chips in the market. Then, three panels of uniformly-distributed SNPs (1K, 5K, and 10K) were selected from the list of 16K common SNPs using the selectSNP package (Wu et al., 2016). The reason for using subsets of uniformly-distributed SNPs in the present study was because they tended to minimize linkage disequilibrium on average, given the number of reference SNPs.

The reference animals for each of the nine pure breeds (not including Brangus) were selected using the 5K SNP panel based on the likelihood approach previously described by He et al. (2018). Briefly speaking, the likelihood that an animal belonged to a specific breed was computed, assuming independent multinomial distributions of the SNP genotypes, computed for each animal. Then, outliers were excluded from each reference population by removing animals with (-2)log(likelihood) exceeding a given cutoff value (which was taken to be two by default). This process excluded 2,170 animals in total, retaining 101,818 “representative” reference animals for the nine purebred cattle breeds. The distributions of (−2)loglikelihoods computed for the animals in the nine pure breeds are shown in Figure S1.

### Admixture Model

Consider *M* SNPs, each having two alleles A and B. The three possible genotypes were coded numerically to be 2 (AA), 1 (AB), and 0 (BB). Let there be *L* reference (or putatively ancestral) populations, and let *q*_*jk*_ be the frequency of allele *A* at the kth SNP in the jth reference population. For a given animal, denote X = [*x*_1_, *x*_2_, …, *x*_*k*_] ′ to be the vector of admixture coefficients, where *x*_*j*_ represents the genomic admixture proportion of this animal of the jth population. Then, weighted allele frequency at SNP *k*, given the allele frequencies and the admixture proportions for each reference population, was computed to be $f_{k}=\overset{L}{\underset{j=1}{\sum }}q_{jk}x_{j}$. Assuming Hardy-Weinberg equilibrium (HWE) at each SNP locus, a genotype, say *g*_*k*_ at locus *k*, is an instance generated with the following probabilities:

(1) $$\begin{array}{l} \mathrm{Pr}\left(g_{k}|f_{k}\right)=\{\begin{array}{ll} f_{k}^{2} & if\;g_{k}=2\\ 2f_{k}\left(1-f_{k}\right) & if\;g_{k}=1\\ {\left(1-f_{k}\right)}^{2\;} & if\;g_{k}=0 \end{array} \end{array}$$

The log-likelihood of all the observed genotypes on this individual was given by:

(2) $$\begin{array}{l} L\left(X\right)=\sum_{i=1}^{M}\ln\left(\mathrm{Pr}\left(g_{k}|f_{k}\right)\;\right) \end{array}$$

The above likelihood (2) can be written as:

(3) $$\begin{array}{l} L\left(X\right)=\sum_{k=1}^{M}\left[g_{i}\ln(f_{k})+(2-\;g_{k})\;\ln\;(1-f_{k}\;)\right]+C \end{array}$$

where $C=\overset{M}{\underset{k=1}{\sum }}\ln\left(\begin{array}{c} 2\\ g_{k} \end{array}\right)$. Our goal was to determine the values for the admixture coefficient vector X = [*x*_1_, *x*_2_, …, *x*_*k*_]′ that maximizes L(X) subject to the constraints *x*_*j*_ ≥ 0 and $\underset{j}{\sum }x_{j}=\;1$.

### Regularized Admixture Model With L1 Norm Penalty

In the ADMIXTURE-L1 model, estimates of sparse solution X of the model (2) were obtained by maximizing the logarithm of likelihood of the data with sparsity enforcing L1-norm penalty on parameters {*x*_*j*_} ( *j* = 1, **⋯, ***k***)** as follows:

(4) $$\begin{array}{l} F\left(X\right)\;≜L\left(X\right)-\left(\overset{k}{\underset{j=1}{\sum }}\lambda |x_{j}|\right), \end{array}$$

where λ(λ > 0) is Lagrange multiplier (i.e., a regularization parameter) that determines the amount of sparsity in *x*_*j*_.

The gradient of L(X ) with respect to *x*_*j*_ were given by

(5) $$\begin{array}{l} {\;\nabla }_{{\;x}_{j}}L\left(X\right)=\sum_{1}^{n}\left[\frac{g_{i}q_{ij}}{\;f_{i}}+\;\frac{\left(2-g_{i}\right)\left(1-q_{ij}\right)}{S\left(X\right)-f_{i}}\right]-\frac{2n}{S\left(X\right)}\; \end{array}$$

where S(X) denotes the sum of the admixture coefficients.

In (4), L(X ) of F(X) is differentiable with respect to $X_{j}.$ Solving (4) is complicated by the non-differentiability of ${|X}_{j}|$ at $X_{j}=0$. We used the subgradient with minimum norm (Bertsekas et al., 2003) of F(X) in (4) as the steepest descent direction and took a step resembling the Newton iteration in this direction with a Hessian approximation to solve the above problem (Gill et al., 1984). Subgradient methods are among the most popular ways for non-differentiable optimization (Bertsekas et al., 2003). More detail on the calculation of the search direction is available in Appendix A.

### Regularized Admixture Model With MCP or SCAD Penalty

In ADMIXTURE-MCP and ADMIXTURE-SCAD, the estimate of sparse solution X of the model (2) is obtained by maximizing the logarithm of likelihood of the data sparsity enforcing non-convex penalty MCP on the parameters {*x*_*j*_} ( *j* = 1, **⋯, ***k***)** as follows:

(6) $$\begin{array}{l} F\left(X\right)\;≜L\left(X\right)-\;\sum_{j=1}^{k}r_{\lambda }\left(\left|x_{j}\right|\right) \end{array}$$

where λ(λ > 0) and $r_{\lambda }\left(\left|x_{j}\right|\right)=\lambda \;\left(\left|x_{j}\right|-\frac{{x_{j}}^{2}}{2\lambda \gamma }\right).{\text{I}}_{\left\{|x_{j}|<\lambda \gamma \right\}}+\frac{\lambda^{2}\gamma }{2}.{\text{I}}_{\left\{|x_{j}|\geq \lambda \gamma \right\}}$ (I_{ϵ}_ = 1 *if ϵ* holds, and I_{ϵ}_ = 0 otherwise).

Given γ > 1, SCAD has

(7) $$\begin{array}{l} r_{\lambda }\left(\left|x_{j}\right|\right)=\lambda |x_{j}|.{\text{I}}_{\left\{|x_{j}|<\lambda \right\}}+\left(\frac{\lambda \gamma }{\gamma -1}\left|x_{j}\right|-\frac{{x_{j}}^{2}+\lambda^{2}}{2\left(\gamma -1\right)}\right).\\ {\text{I}}_{\left\{\lambda <|x_{j}|<\lambda \gamma \right\}}+\frac{\lambda^{2}\left(\gamma +1\right)}{2}.{\text{I}}_{\left\{|x_{j}|\geq \lambda \gamma \right\}} \end{array}$$

In the above, γ is the concavity parameter of MCP or SCAD, which essentially characterizes the concavity of the MCP or SCAD regularizer: A larger γ implies that the regularizer is less concave. In this paper, we let γ = 3 as usual. Please refer to Appendix A for obtaining the subgradient of *r*_λ_(|*x*_*j*_|) and Appendix B for computing GBC using Algorithm 1 by just replacing the subgradient of |*x*_*j*_| with the subgradient of *r*_λ_(|*x*_*j*_| ).

## Results and Discussion

### Determining Optimal Values for the Regularization Parameter **λ**

**Figure 1:**
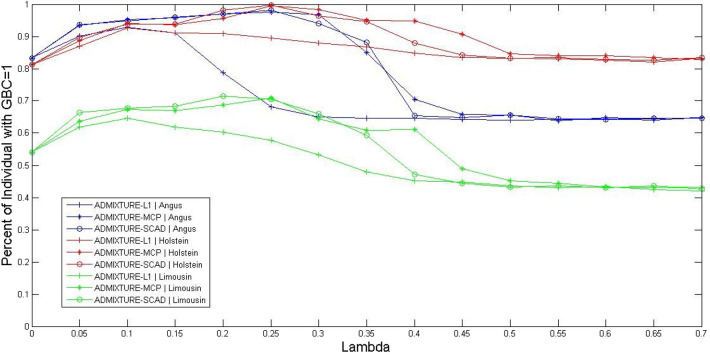
Percent of individuals with GBC=1 obtained by the three regularized ADMIXTURE methods, each with a varying value for the regulation parameter lambda (λ). Curves were extracted from the surfaces in this figure by fixing the GBC =1 for ADMIXTURE-L1, ADMIXTURE-MCP, and ADMIXTURE-SCAD in Angus, Holstein, and Limousin, respectively.

The optimal values for the parameter λ of the three sparsely regularized admixture models were obtained using three-fold cross-validation, based on the 5K SNP panel, and illustrated in three cattle breeds (Angus, Holstein, and Limousine). The non-regularized admixture model served as the baseline model for comparison because it was equivalent to ADMIXTURE-L1 with λ = 0. Briefly, all the animals for each breed were randomly split into three subsets. Then, the animals in two subsets were combined and used as the reference population for estimating the allele frequencies of SNPs in the 5K panel. The third subset was used as the testing set, in which GBC was computed for each animal. The procedure rotated three times so that each subset was used for testing once and only once. The percentage of animals with GBC = 1 for their respective breeds was computed for each of the three sparsely regularized admixture models under varied settings for the regularization parameter λ. Then, the optimal values of regularization parameter λ were taken as such that each sparsely regularized admixture model gave a higher percentage of purebred animals with 100% GBC of their respective breeds than the non-regularized ADMIXTURE (λ = 0). By this criterion, the range of optimal values of λ for the three regularized admixture models appeared to be 0 < λ < 0.60 for Holstein, 0 < λ < 0.36 for Angus, and 0 < λ < 0.30 for Limusine (see Figure 1). In Holstein, the maximal percentage of individual animals with GBC =1 was 92.7% (ADMIXTURE-L1 with λ = 0.1), 99.5% (ADMIXTURE-MCP with λ = 0.25), and 99.7% (ADMIXTURE-SCAD with λ = 0.25). In Angus, the maximum percentage of individuals with GBC = 1 obtained using the regularized admixture models was 92.9% for ADMIXTURE-L1 with λ = 0.1, 97.6% for ADMIXTURE-MCP with λ = 0.25, and 98.2% for ADMIXTURE-SCAD with λ = 0.25. In Limousine, the maximal percentage of individuals with GBC =1 was relatively lower, which was 64.6% (ADMIXTURE-L1 with λ = 0.1), 70.9% (ADMIXTURE-MCP with λ= 0.25), and 71.4% (ADMIXTURE-SCAD with λ = 0.20). We, therefore, decided to take λ = 0.1 for Admixture-L1, and λ = 0.25 for Admixture-MCP and Admixture-SCAD to estimate GBC in the following analyses.

### Estimated Genomic Breed Composition for Purebred Animals

With the optimal λ values given to the regularized models and λ = 0 for the non-regularized model, GBC was estimated for animals in each of the nine pure breeds using the four statistical models. In Table 2 are the percentages of animals by the ranges of estimated GBC obtained using the four models with the 16K SNP panel for Angus, Holstein, and Limousine, respectively. Estimated GBC for these three breeds using all the four SNP (1K, 5K, 10K, and 16K) are shown in Tables S1– S3. Furthermore, estimated GBC for all the six breeds (also including Brahman, Hereford, Jersey, Shorthorn, Simmental, and Wagyu breeds) using the 5K SNP panel are shown in Tables S2–S5. Hereafter, the percent of animals with GBC = 1 in each breed was taken empirically to be the power for the identification of purebred animals, though this criterion was stringent.

**Table 2:** Percent (%) of animals by categories of estimated GBC obtained using four statistical models with the 16K SNP panel in Angus (A), Holstein (H), and Limousine (L).

**GBC**	**Admixture**			**Admixture-L1**			**Admixture-MCP**			**Admixture-SCAD**		
	**A**	**H**	**L**	**A**	**H**	**L**	**A**	**H**	**L**	**A**	**H**	**L**
1	69.6	70.7	47.4	94.1	97.7	65.1	98.6	99.2	72.5	96.5	99.6	70.9
[0.9, 1)	18.9	19.5	9.4	3.3	1.2	6.7	0.4	0.3	4.4	2.3	0.1	4.3
[0.8, 0.9)	8.5	7.0	9.4	1.5	1.0	5.8	0.5	0.4	3.6	0.4	0.1	4.5
[0.7, 0.8)	1.8	2.4	9.2	0.5	0.1	8.7	0.1	0.0	5.0	0.2	0.0	6.1
[0.6, 0.7)	0.4	0.2	13.5	0.2	0.0	6.9	0.2	0.0	5.5	0.2	0.0	7.2
[0.5, 0.6)	0.3	0.0	6.2	0.1	0.0	2.8	0.1	0.0	4.4	0.1	0.0	3.5
[0.5, 0.4)	0.2	0.0	2.6	0.1	0.0	2.0	0.0	0.0	1.8	0.1	0.0	1.1
[0.4, 0.3)	0.1	0.0	1.2	0.1	0.0	0.9	0.0	0.0	1.2	0.0	0.0	0.8
[0.3, 0.2)	0.1	0.0	0.5	0.1	0.0	0.8	0.0	0.0	0.8	0.0	0.0	0.4
[0.2, 0.1)	0.0	0.0	0.4	0.0	0.0	0.2	0.0	0.0	0.4	0.0	0.0	0.3
[0.1, 0)	0.0	0.0	0.0	0.0	0.0	0.0	0.0	0.0	0.0	0.0	0.0	0.0

*ADMIXTURE, non-regularized admixture model (λ= 0); ADMIXTURE-L1, admixture model with L1 norm penalty (λ= 0.1); ADMIXTURE-MCP, admixture model with MCP penalty (λ= 0.25); ADMIXTURE-SCAD, admixture model with SCAD penalty (λ= 0.25)*.

The power of identifying purebred animals varied with the size of SNP panels. The 1K SNP panel had the highest power for identifying purebred animals in most of the nine breeds, e.g., Angus and Limous, and the power of identifying purebred animals decreased as the SNP panel size increased (Table S1). In Holstein, the 1K SNP panel had either greater or approximately comparable power as the 16K SNP panel (Table S1). The loss in power as the panel size increased was large with the non-regularized model but very slightly with the three regularized models. A possible reason is the following. The admixture assumed that all SNP loci were independent in the likelihood. However, this assumption did not hold precisely in reality due to linkage disequilibrium (LD) between SNPs. With uniformly-distributed SNPs, we found that the 1K SNP panel had the smallest LD between SNPs, compared to the larger SNP panels. Thus, the 1K SNP panel gave more accurate likelihood values computed for these animals than those obtained with larger SNP panels, subsequently leading to the highest power for identifying purebred animals. Nevertheless, the models with regularization seemed to be more robust to the violation of the model assumption about the independence of SNPs than the non-regularized model.

Of the four admixture models, the regularized admixture models had higher power in the identification of purebred animals than the non-regularized admixture model. With the 16K panel, for example, the percentage of animals with Angus GBC =1 was 69.6% with the non-regularized admixture model, and it was substantially higher (94.1–97.3%) with the three regularized models (Table 2). Similar trends were observed in all the other breeds (Tables S1–S7). Concerning the three models with regularization, the two models with non-convex penalties (ADMIXTURE-MCP and ADMIXTURE-SCAD) had a higher power for identifying purebred animals than the one with the L1 norm penalty (ADMIXTURE-L1).

**Figure 2:**
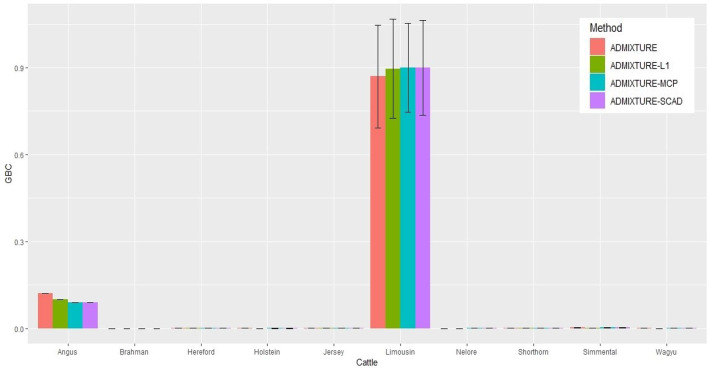
Histogram of the means of estimated GBC for 5,041 Limousin animals, obtained using four statistical models, respectively. Bar plot of the mean GBC across the 10 breeds, which were estimated by ADMIXUTUR ADMIXUTURE-L1 (λ = 0.1), ADMIXUTURE-MCP (λ = 0.25), and ADMIXUTURE-SCAD (λ = 0.25) using 5K SNP panel. Standard deviations (SD) is abled on the bar of Limousin.

The identification power of purebred animals varied drastically with the nine breeds. The percent of animals with GBC = 1 was the lowest (47.4–74.4%) in Limousine (Table 2) and the highest (99.7–100%) in Brahman (Table 2 and Tables S1–S7). Because Brahman was the only *indicus* cattle breed, which had distant relationships with the *taurus* cattle breeds, the power of identifying purebred Brahman cattle was thus the highest. For the remaining seven breeds, the percent of animals with GBC =1 obtained using the three regularized admixture models with the 5K SNP panel was high in Angus (93.3–98.4%) (Table S1), Hereford (97.6–99.8%) (Tables S5–S7), Holstein (93.2–99.7%) (Table S1), Jersey (97.4–99.3%) (Tables S5–S7), and Wagyu (95.1–98.8%) (Tables S5–S7), but was it was relatively low in Shorthorn (79.5–83.7%) and Simmental (60.1–65.1%) (Tables S5–S7). There were mainly two main reasons for the low power of purebred identification in Limousine and Simmental. In Limousine, for example, there was an unignorable number of the “Limousine” animals, which were possibly “progressive” crosses of Limousine with Angus arity of Limousine cattle with Angus (Figure 2) and not excluded when applying the cutoff of (−2)loglikelihood > 2 during the data cleaning (Figure S1F). Thus, the estimated GBC for these “Limousine” animals showed an unignorable portion of Angus GBC (Figure 2). The three regularized admixture models improved the power substantially but limited by the portion of “progressive” crosses of Limousine. A similar situation was observed with Simmental cattle as well.

### Estimation of GBC for Composite Animals

The four admixture models were also used to estimate GBC for the 3,605 Brangus animals. This composite beef breed was developed to utilize the superior traits of Angus and Brahman cattle. For official registration, a Brangus animal is expected to be genetically stabilized at 3/8 Brahman and 5/8 Angus, solid black or red, and polled, and both sire and dam must be recorded with the International Brangus Breeders Association (IBBA). Unlike estimating GBC for a purebred animal, our interest for a composite animal was to know how much of its genome was inherited from each of its ancestral breeds.

**Figure 3:**
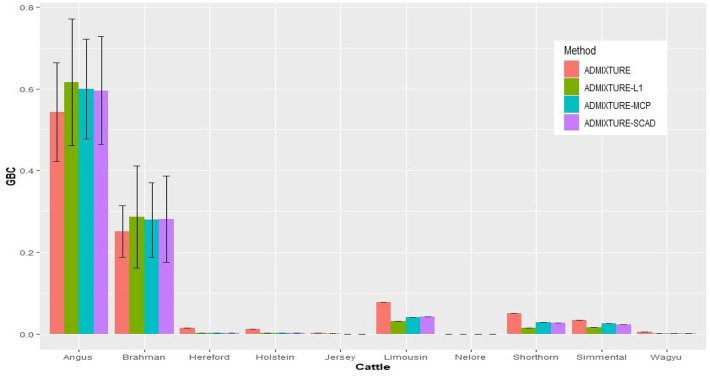
Histogram of the means of estimated GBC for 3,605 Brangus(0.625 Angus, 0.375 Brahman) obtained the four statistical models, respectively. Bar plot of the mean GBC across the ten breeds, which were estimated by ADMIXUTUR, ADMIXUTURE-L1 (λ = 0.1), ADMIXUTURE-MCP (λ = 0.25), and ADMIXUTURE-SCAD (λ = 0.25) using 5K SNP panel. Standard deviations (SD) were abled on the Angus and Brahman bars.

With the nine reference populations and the 5K SNP panel, small admixture coefficients showed up for non-ancestral breeds, such as Hereford, Limousine, Shorthorn, and Simental, in addition to the two large admixture components for the two ancestral breeds (Figure 3). Because of these non-zero GBC for non-ancestral breeds, the estimated GBC of Brangus pertaining to the two ancestral breeds (Angus and Brahman) were underestimated, and these two ancestral admixture components did not add up to 1 (Table 3). For example, based on the non-regularized ADMIXTURE model, these Brangus were on average 54.3% Angus and 25.1% Brahman. The three regularized admixture models elevated the estimated GBC for the two ancestral breeds, possibly owing to the penalties imposed on small GBC components of non-ancestral breeds, but the estimated GBC for Angus (59.5–61.5%) and Brahman (27.9–28.6%) were still were under-estimated, and they did not add up to 1 (Table 3). It is a well-known fact that the Brangus are descendants of Angus and Brahman. Hence, one can reasonably compute the GBC of Angus and Brahman, respectively, as relative ratios of admixture components corresponding to these two breeds only while ignoring estimated GBC for the remaining breeds. The latter can be understood as the conditional probability of GBC of the two ancestral breeds for Brangus, given the probability that Angus and Brahman are their ancestors. The “conditionally” estimated GBC for these Brangus using the non-regularized admixture model was on average 68.3% Angus and 31.7% Brahman, whereas, with the three regularized admixture models, average estimated GBC was 67.9–68.2% Angus and 31.8–32.1% Brahman (Table 3). Alternatively, GBC for these Brangus was estimated by including only the two ancestral breeds in the reference. With the latter approach, the average estimated GBC for Brangus was 71.1% Angus and 28.9% Brahman based on the non-regularized admixture model and 74.6–77.1% Angus and 22.9–25.4% Brahman based on the three regularized admixture models (Table 3).

**Table 3:** Percent (%) of animals by categories of estimated GBC obtained using four statistical models in Brangus.

**Model**	**Nine-reference breeds**				**Two-reference (ancestral) breeds**			
	**Angus**		**Brahman**		**Angus**		**Brahman**	
	**Mean^a^**	***SD***	**Mean**	***SD***	**Mean**	***SD***	**Mean**	***SD***
ADMIXTURE	54.3 (68.3)	11.9	25.1 (31.7)	6.31	71.1	6.70	28.9	6.70
ADMIXTURE-L1	61.5 (68.2)	15.6	28.6 (31.8)	12.1	77.1	8.70	22.9	8.70
ADMIXTURE-MCP	59.8 (68.1)	12.9	27.9 (31.9)	9.1	74.6	7.10	25.4	7.10
ADMIXTURE-SCAD	59.5 (67.9)	13.1	28.1 (32.1)	10.4	75.3	7.50	24.7	7.50

a *In the brackets are the relative GBC ratio of Angus and Brahman origin only, respectively, computed with nine reference breeds*.

**Figure 4:**
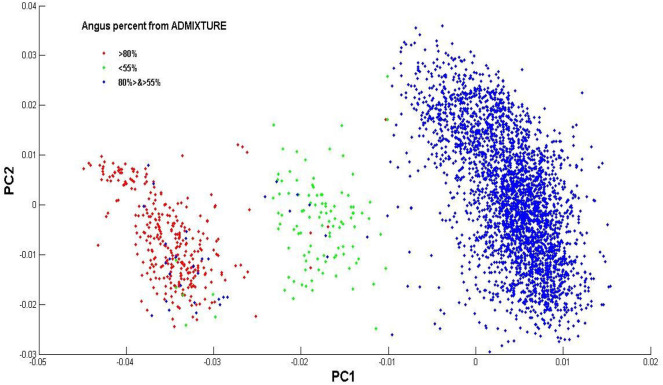
Population distribution across the first (PC1) and second principal component (PC2) on the genotype data of the Brangus individuals. Animals are labels based on their Angus percent of GBC estimated by ADMIXTURE.

The estimated Angus composition in these Brangus animals, as obtained using the four models, were presumably higher than the pedigree-expected Angus ratio of 62.5%. There were possibly two reasons for the elevated Angus GBC. Firstly, the Brangus have been selected for traits with which Angus has advantages. Hence, the selection, in turn, could shift allelic frequencies more toward the Angus origin. Secondly, there was a mixture of UltraBlack animals in this Brangus dataset. A King-robus principal component analysis (PCA) based on the genotypes of the 3,605 Brangus was conducted to infer the genetic relationships of these Brangus animals using the King-robus software (Manichaikul et al., 2010). The first principal component (PC1) and the second principal component (PC2) described 25.1 and 11.6%, respectively, of the total variation of Angus GBC in this Brangus population. Three clusters were identified in Figure 4, which suggested population stratification of Brangus that varied in their genomic composition for Angus. The majority (~86%) of these Brangus cattle were 55–80% Angus. For the remaining Brangus cattle, around 4% of animals were < 55% Angus, and around 10% of animals were >80% Angus. The Brangus cattle having >80% Angus genomic component were mostly “Ultrablack” (UB) animals. In October 2005, the International Brangus Breeders Association (IBBA) board of directors approved the creation of the “Ultrablack” program to take advantage of the strengths of the Brangus and Angus. A 1/2 “Ultrablack” animals (i.e., the progeny produced from mating a registered Brangus to a registered Angus) were, on average, 81.25% Angus. Finally, these sparsely-regularized models consistently produced larger estimated GBC than the non-regularized model, which might be an indication of possible estimation errors. The true GBCs of these Brangus animals, however, were unknown.

Finally, two assumptions under the present models are worth discussion. First, it was assumed that each reference population comprised samples of purebred animals only. This assumption, however, can be violated in reality because a low level of introgression in the reference samples can occur. For example, Brahman cattle carry an average composition of 91% Bos indicus and 9% Bos taurus (O'Brien et al., 2015). Some of the taurine genome retained in Brahman even resulted from recent artificial selection (Fortes et al., 2013). Clustering errors indistinguishable from the admixture methods occur when ghost admixture (i.e., introgression from an unsampled population) or recent bottlenecks are embedded into the demographic history of an analyzed population (Lawson et al., 2018). Nevertheless, this assumption was taken approximately for the convenience of modeling and computation. We also observed that, given a significant number of animals in a reference population, the deviation in estimated allelic frequencies for this reference population due to the mixture of a tiny portion of cross-bred animals tended to ignorable. Therefore, its impact on the estimated GBC of the test animals also tended to be trivial as well. In the Brahman population, for example, there are 25 crossbred progenies of Brahman, which were excluded from the reference population in the present study. But including them in the reference had very little impact on the estimated allelic frequencies and the estimated GBC of the test animals in the present study.

Secondly, the present admixture models assumed that the allele frequencies of the ancestral breeds are known and are estimated a prior, which differed from the unsupervised model-based clustering algorithms. The latter was originally conceived to not only estimate ancestry in admixed individuals but also to study the trajectory of divergence between ancestral populations that produced the empirical data. This is important because modern-day breeds of cattle—especially Bos taurus breeds—were formed quite recently (i.e., in an evolutionary scale) from mixtures of previously geographically isolated lineages that were only moderately divergent (FST < 0.10), and are not necessarily pure distinct lineages from a population genetics stand point. Assuming fixed allele frequencies for ancestral ignore the trajectory of genetic characteristics of ancestral populations over time, but it simplifies the computing in practice. This is particularly advantageous with the proposed sparsely-regularized admixture models, which are often more computationally intensive than the non-regularized admixture models. Finally, some methods can even accommodate complex admixtures, such as support vector machines (Haasl et al., 2013; Durand et al., 2014). Comparison of our methods with support vector machines was not evaluated in the present study but can be of interest for future studies.

## Conclusion

Estimated GBC for purebred animals is complicated by the presence of small admixture components assigned to non-ancestral breeds due to the genomic similarities. Thus, not all purebred animals have 100% GBC for their respective breed categories, leading to an increased false-negative rate for pure-breed identification. Otherwise, a lower cutoff of estimated GBC for purebred animals needs to be used instead, which, however, is arbitrary. Our results showed that the use of sparse regularization in the admixture models with appropriately-chose values of λ effectively shrank non-ancestral GBC estimates toward zero, therefore reducing the false-negative rate and at the same time increasing the identification power of purebred animals. Of the three sparse regularized admixture models, the two models with nonconvex penalties (ADMIXTURE-MCP and ADMIXTURE-SCAD) outperformed the admixture model with L1 norm penalty (ADMIXTURE-L1).

The power of breed identification of purebred animals varied with reference SNP panels used in the non-regularized admixture model. The 1K panel giving the greatest power in most breeds because it had the smallest average LD between SNPs, which approximately satisfied the model assumption about the independence of SNPs. Therefore, the computed likelihood values using the 1K panel are more accurate than larger panels (5K, 10, and 16K). Nevertheless, the three regularized admixture models were more robust to the violation of model assumption for SNP independence than the non-regularized admixture model when estimating GBC using various SNP panels, because the power of purebred identification with the regularized admixture model decreased at a considerably slower rate than the non-regularized admixture model as the SNP panel sizes increased. As a rule of thumb, a cutoff of GBC for pure-breed identification is recommended to be 95% for the non-regularized admixture model and between 0.98 and 0.99 for regularized admixture models, assuming no significant population stratification and no significant genomic correlations between the reference breeds.

For composite animals, the three admixture models with sparse regularization tended to produce larger GBC for these Brangus animals than the non-regularized admixture model, which possibly indicated the presence of estimation bias with the regularized models. While imposing sparse regularization on estimated GBC is favorable for reducing false-negative error rate when identifying purebred animals, it can lead to bias in estimated GBC for crossbred or composite animals, in particular when dynamic segregation was still going on. Hence, the utility of regularized admixture models for estimating GBC in composite animals needs to be taken with caution and the results need to be checked against those obtained using non-regularized admixture models.

Finally, a software package that implements the admixture models with regularization is made available for non-commercial use (The web link will be provided once the paper is accepted).

## Data Availability Statement

The supplementary results, four reference SNP panels, namely 1K, 5K, 10K, and 16K (actually 14K after data cleaning), and two example GGP 50K genotype files (each with 1000 animals) are available at the following link: https://drive.google.com/open?id=1qfwyK-Qpp4SvRcj23w-q7bYLE3MgQd1L. For the protection of commercial confidence, all reference SNPs, breeds, and animal IDs are re-coded anonymously.

## Ethics Statement

Ethical review and approval were not required for the study because the genotypes were extracted from the data repositories of Neogen genotyping laboratories. All the cattle samples (hair, blood and ear tags) used for genotyping are collected based on routine procedures for commercial selection purposes.

## Author Contributions

YW, XL-W, and GR conceived this study, in discussion with ZB, RT, and SB. YW and XL-W drafted and revised the manuscripts. YW and ZL conducted the data analysis. All the authors read and approved this manuscript.

## Conflict of Interest

X-LW, RT, and SB were employed by the company Neogen. The remaining authors declare that the research was conducted in the absence of any commercial or financial relationships that could be construed as a potential conflict of interest.
