# Supplementary material for: Estimation of Genomic Breed Composition for Purebred and Crossbred Animals Using Sparsely Regularized Admixture Models
Source: Front Genet. 2020 Jun 11;11:576. doi: 10.3389/fgene.2020.00576 (PMC7300184; doi:10.3389/fgene.2020.00576)
Supplement: Supplementary file 1 [file Data_Sheet_1.docx]

**Table S1．Percent (%) of animals by categories of estimated GBC obtained using ADMIXTURE with5 K SNP panel in Brahman, Hereford, Jersey, Shorthorn, Simmental, and Wagyu.**

|  |  | ADMIXTURE | | | | | |  | | | | |  | |  | |
| --- | --- | --- | --- | --- | --- | --- | --- | --- | --- | --- | --- | --- | --- | --- | --- | --- |
|  | GBC | Brahman | Hereford | | Jersey | Shorthorn | | | Simmental | | Wagyu | |  | | |  |
|  | =1 | 99.7 | 84.2 | 89.0 | | | 75.6 | | | 49.8 | | 83.9 | |  |  |  |
|  | [0.9,1) | 0.00 | 13.4 | 6.74 | | | 3.62 | | | 9.65 | | 8.35 | |  |  |  |
|  | [0.8,0.9) | 0.00 | 1.85 | 2.75 | | | 2.20 | | | 8.85 | | 4.57 | |  |  |  |
|  | [0.7,0.8) | 0.20 | 0.33 | 0.91 | | | 1.47 | | | 10.5 | | 1.90 | |  |  |  |
|  | [0.6,0.7) | 0.00 | 0.00 | 0.23 | | | 6.25 | | | 6.11 | | 0.92 | |  |  |  |
|  | [0.5,0.6) | 0.00 | 0.00 | 0.25 | | | 9.77 | | | 3.99 | | 0.29 | |  |  |  |
|  | [0.5,0.4) | 0.00 | 0.00 | 0.04 | | | 0.49 | | | 3.14 | | 0.00 | |  |  |  |
|  | [0.4,0.3) | 0.00 | 0.00 | 0.00 | | | 0.24 | | | 2.48 | | 0.00 | |  |  |  |
|  | [0.3,0.2) | 0.00 | 0.00 | 0.00 | | | 0.00 | | | 2.01 | | 0.00 | |  |  |  |
|  | [0.2,0.1) | 0.00 | 0.00 | 0.00 | | | 0.00 | | | 1.07 | | 0.00 | |  |  |  |
|  | [0.1, 0) | 0.00 | 0.00 | 0.00 | | | 0.00 | | | 2.24 | | 0.00 | |  |  |  |

ADMIXTURE = non-regularized admixture model (λ=0).

**Table S2．Percent (%) of animals by categories of estimated GBC obtained using ADMIXTURE-L1 with5 K SNP panel in Brahman, Hereford, Jersey, Shorthorn, Simmental, and Wagyu.**

|  |  | ADMIXTURE-L1 | | | | | |  | | | | |  | |  | |
| --- | --- | --- | --- | --- | --- | --- | --- | --- | --- | --- | --- | --- | --- | --- | --- | --- |
|  | GBC | Brahman | Hereford | | Jersey | Shorthorn | | | Simmental | | Wagyu | |  | | |  |
|  | =1 | 99.7 | 97.6 | 97.4 | | | 79.5 | | | 60.1 | | 95.1 | |  |  |  |
|  | [0.9,1) | 0.00 | 1.44 | 1.15 | | | 3.23 | | | 9.98 | | 1.95 | |  |  |  |
|  | [0.8,0.9) | 0.20 | 0.70 | 0.89 | | | 2.43 | | | 6.08 | | 1.87 | |  |  |  |
|  | [0.7,0.8) | 0.00 | 0.04 | 0.21 | | | 2.87 | | | 6.47 | | 0.66 | |  |  |  |
|  | [0.6,0.7) | 0.00 | 0.04 | 0.17 | | | 8.04 | | | 3.37 | | 0.30 | |  |  |  |
|  | [0.5,0.6) | 0.00 | 0.00 | 0.08 | | | 3.20 | | | 2.05 | | 0.02 | |  |  |  |
|  | [0.5,0.4) | 0.00 | 0.00 | 0.03 | | | 0.41 | | | 1.86 | | 0.00 | |  |  |  |
|  | [0.4,0.3) | 0.00 | 0.00 | 0.00 | | | 0.16 | | | 2.53 | | 0.00 | |  |  |  |
|  | [0.3,0.2) | 0.00 | 0.00 | 0.00 | | | 0.00 | | | 2.02 | | 0.00 | |  |  |  |
|  | [0.2,0.1) | 0.00 | 0.00 | 0.00 | | | 0.00 | | | 2.01 | | 0.00 | |  |  |  |
|  | [0.1, 0) | 0.00 | 0.00 | 0.00 | | | 0.00 | | | 1.50 | | 0.00 | |  |  |  |

ADMIXTURE-L1 = admixture model with L1 norm penalty (λ=0.1).

**Table S3．Percent (%) of animals by categories of estimated GBC obtained using ADMIXTURE-MCP with5 K SNP panel in Brahman, Hereford, Jersey, Shorthorn, Simmental, and Wagyu.**

|  |  | ADMIXTURE-MCP | | | | | |  | | | | |  | |  | |
| --- | --- | --- | --- | --- | --- | --- | --- | --- | --- | --- | --- | --- | --- | --- | --- | --- |
|  | GBC | Brahman | Hereford | | Jersey | Shorthorn | | | Simmental | | Wagyu | |  | | |  |
|  | =1 | 99.7 | 99.7 | 99.2 | | | 83.0 | | | 65.1 | | 98.6 | |  |  |  |
|  | [0.9,1) | 0.00 | 0.08 | 0.28 | | | 3.47 | | | 6.63 | | 0.62 | |  |  |  |
|  | [0.8,0.9) | 0.20 | 0.08 | 0.17 | | | 1.69 | | | 4.20 | | 0.34 | |  |  |  |
|  | [0.7,0.8) | 0.00 | 0.04 | 0.11 | | | 2.95 | | | 4.59 | | 0.26 | |  |  |  |
|  | [0.6,0.7) | 0.00 | 0.00 | 0.12 | | | 4.92 | | | 3.64 | | 0.08 | |  |  |  |
|  | [0.5,0.6) | 0.00 | 0.00 | 0.07 | | | 3.03 | | | 3.29 | | 0.02 | |  |  |  |
|  | [0.5,0.4) | 0.00 | 0.00 | 0.01 | | | 0.57 | | | 2.48 | | 0.00 | |  |  |  |
|  | [0.4,0.3) | 0.00 | 0.00 | 0.00 | | | 0.16 | | | 2.21 | | 0.00 | |  |  |  |
|  | [0.3,0.2) | 0.00 | 0.00 | 0.00 | | | 0.00 | | | 1.98 | | 0.00 | |  |  |  |
|  | [0.2,0.1) | 0.00 | 0.00 | 0.00 | | | 0.00 | | | 2.48 | | 0.00 | |  |  |  |
|  | [0.1, 0) | 0.00 | 0.00 | 0.00 | | | 0.00 | | | 1.31 | | 0.00 | |  |  |  |

ADMIXTURE-MCP = admixture model with MCP penalty (λ=0.25).

**Table S4．Percent (%) of animals by categories of estimated GBC obtained using ADMIXTURE-SCAD with5 K SNP panel in Brahman, Hereford, Jersey, Shorthorn, Simmental, and Wagyu.**

|  |  | ADMIXTURE-SCAD | | | | | |  | | | | |  | |  | |
| --- | --- | --- | --- | --- | --- | --- | --- | --- | --- | --- | --- | --- | --- | --- | --- | --- |
|  | GBC | Brahman | Hereford | | Jersey | Shorthorn | | | Simmental | | Wagyu | |  | | |  |
|  | =1 | 1.00 | 99.8 | 99.3 | | | 83.7 | | | 63.9 | | 98.8 | |  |  |  |
|  | [0.9,1) | 0.00 | 0.04 | 0.19 | | | 4.54 | | | 4.41 | | 0.66 | |  |  |  |
|  | [0.8,0.9) | 0.00 | 0.04 | 0.20 | | | 2.59 | | | 4.42 | | 0.26 | |  |  |  |
|  | [0.7,0.8) | 0.00 | 0.04 | 0.08 | | | 2.62 | | | 3.62 | | 0.13 | |  |  |  |
|  | [0.6,0.7) | 0.00 | 0.00 | 0.04 | | | 3.28 | | | 3.20 | | 0.07 | |  |  |  |
|  | [0.5,0.6) | 0.00 | 0.00 | 0.01 | | | 2.71 | | | 2.59 | | 0.08 | |  |  |  |
|  | [0.5,0.4) | 0.00 | 0.00 | 0.00 | | | 0.08 | | | 1.81 | | 0.00 | |  |  |  |
|  | [0.4,0.3) | 0.00 | 0.00 | 0.00 | | | 0.12 | | | 2.05 | | 0.00 | |  |  |  |
|  | [0.3,0.2) | 0.00 | 0.00 | 0.00 | | | 0.00 | | | 2.25 | | 0.00 | |  |  |  |
|  | [0.2,0.1) | 0.00 | 0.00 | 0.00 | | | 0.00 | | | 1.53 | | 0.00 | |  |  |  |
|  | [0.1, 0) | 0.00 | 0.00 | 0.00 | | | 0.00 | | | 9.59 | | 0.00 | |  |  |  |

ADMIXTURE-SCAD = admixture model with SCAD penalty (λ=0.25).

**Figure S1 :Distribution of (-2)loglikehood computed for Angus (A), Brahman (B), Hereford (C), Holstein (D), Jersey (E), Limousin (F), Shorthorn (G), Simmental (H), and Wagyu (I).**


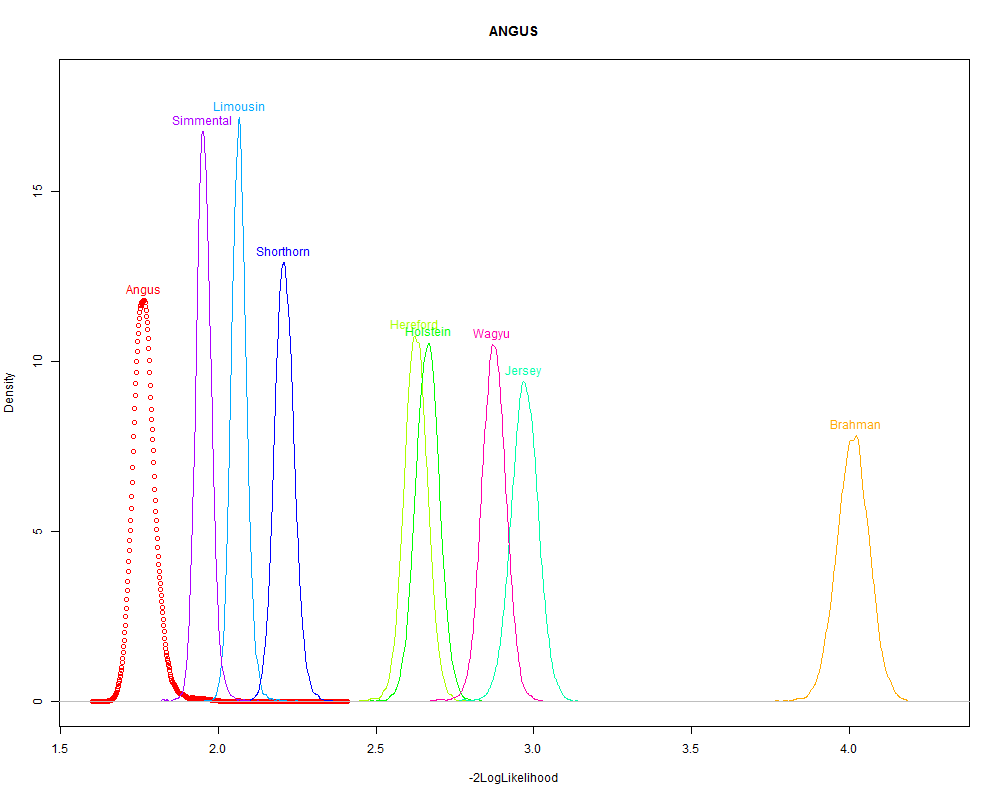


A


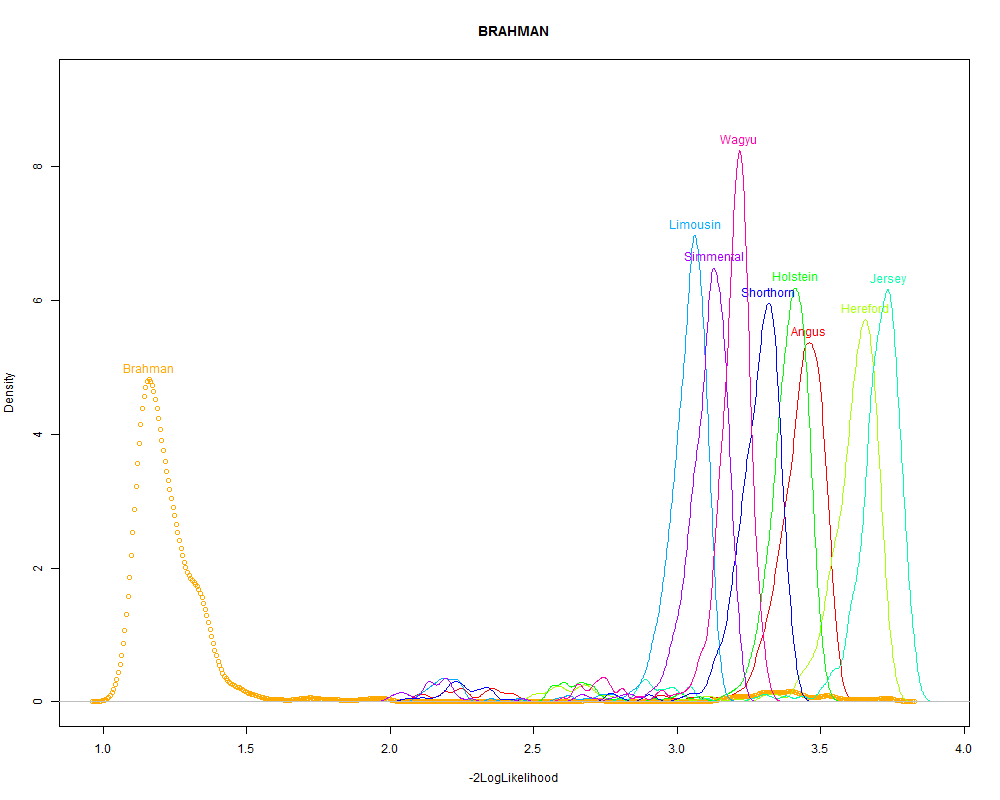


B


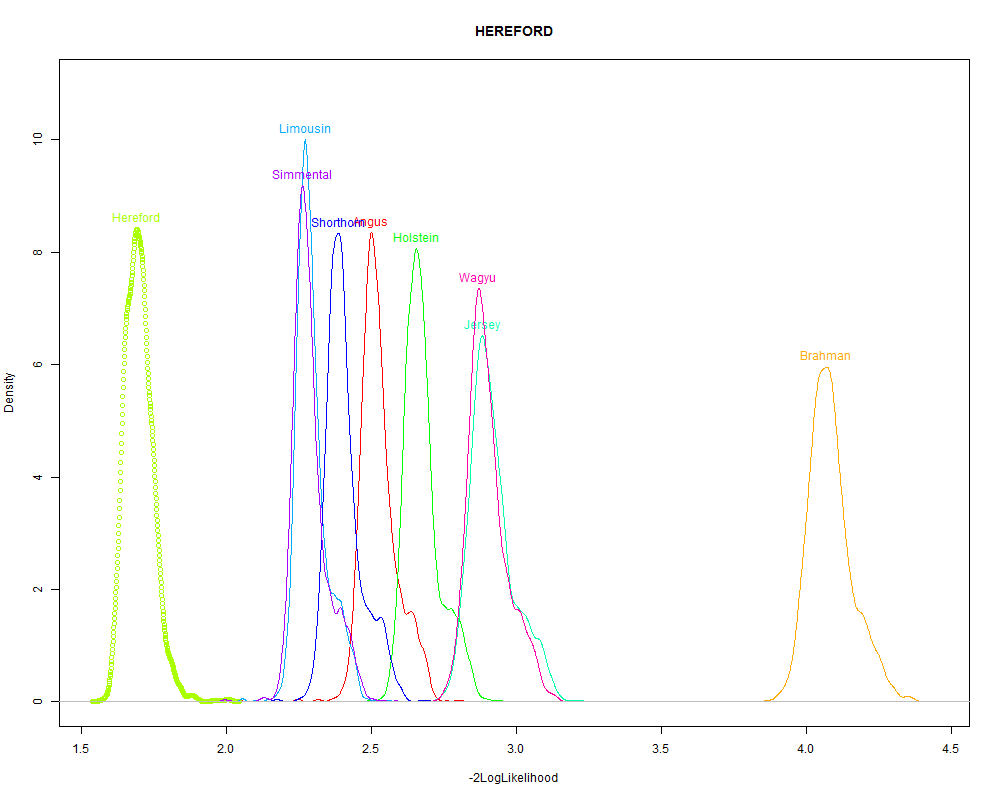


C


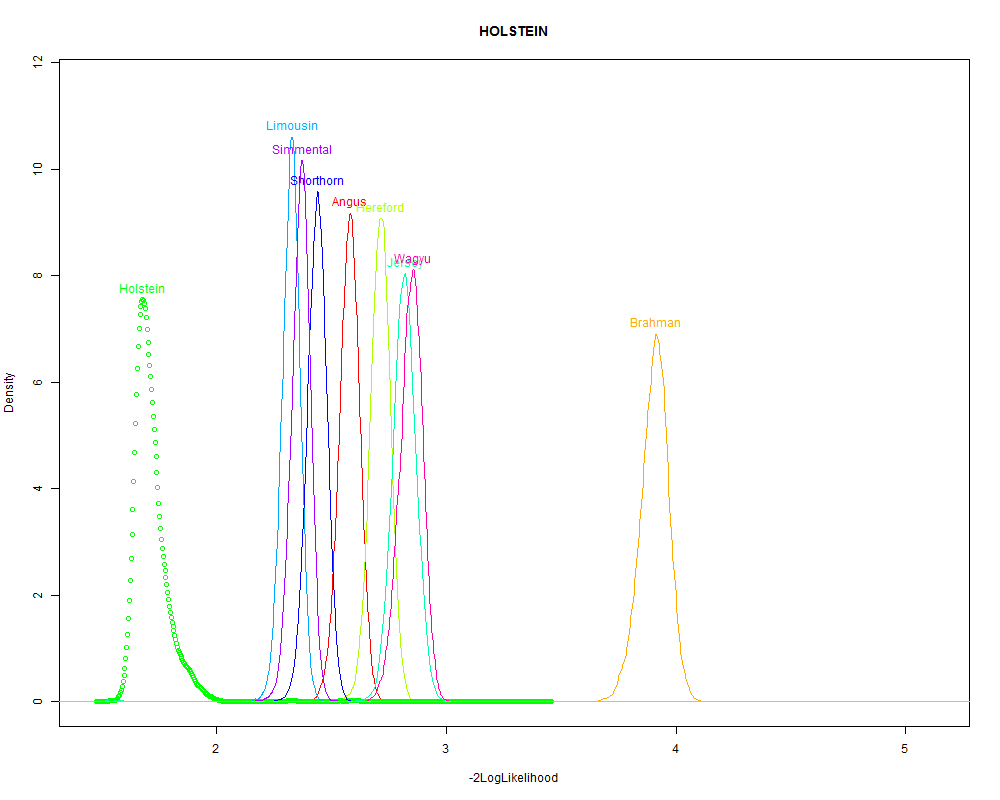


D


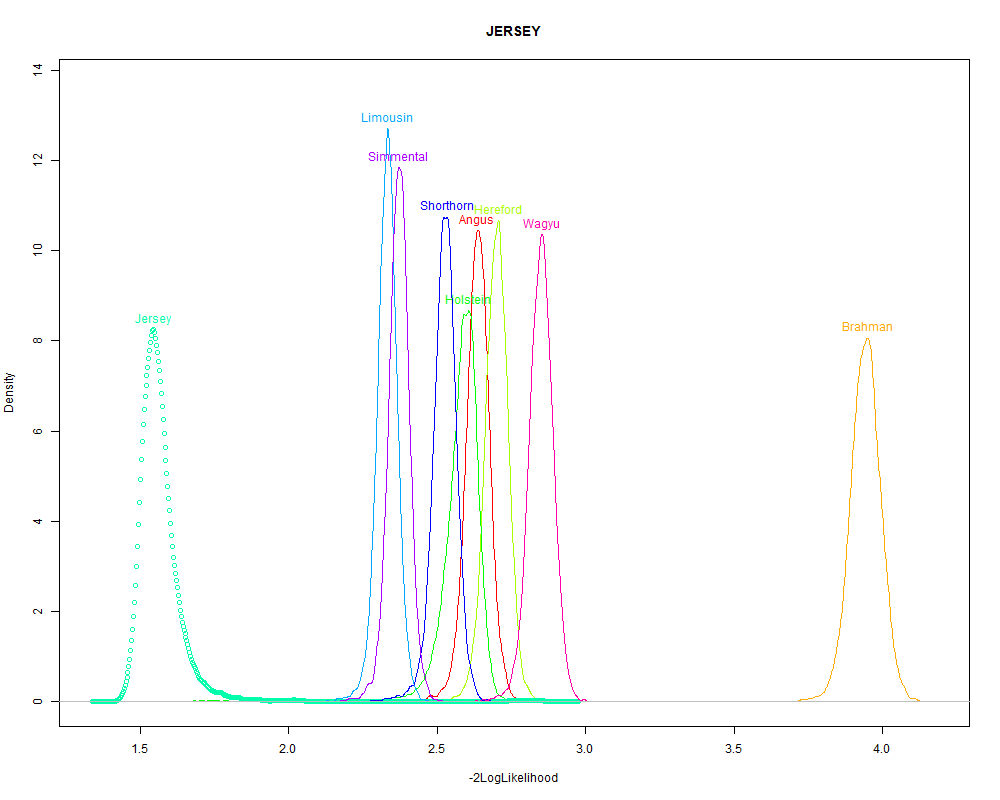


E


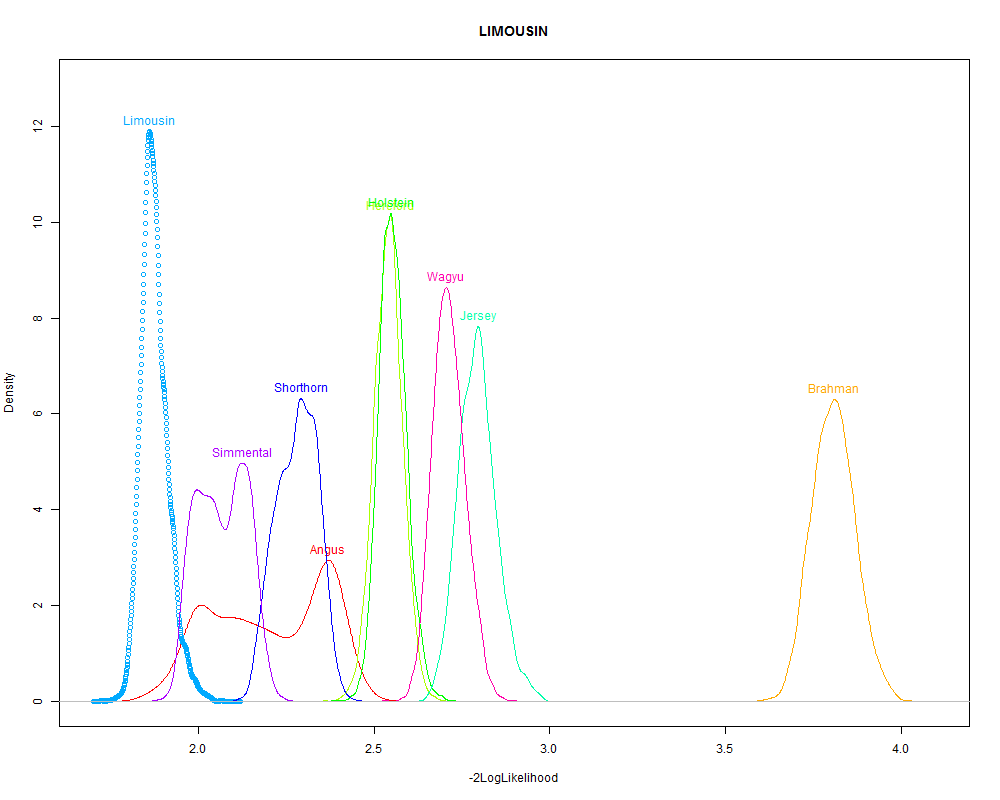


F


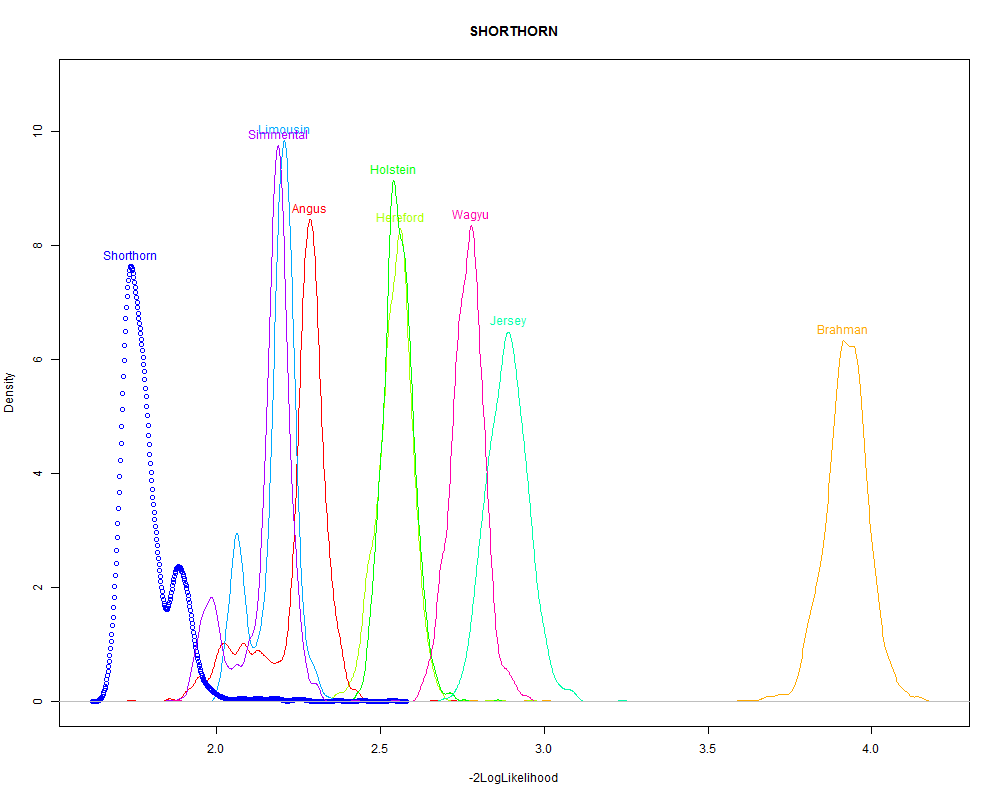


G


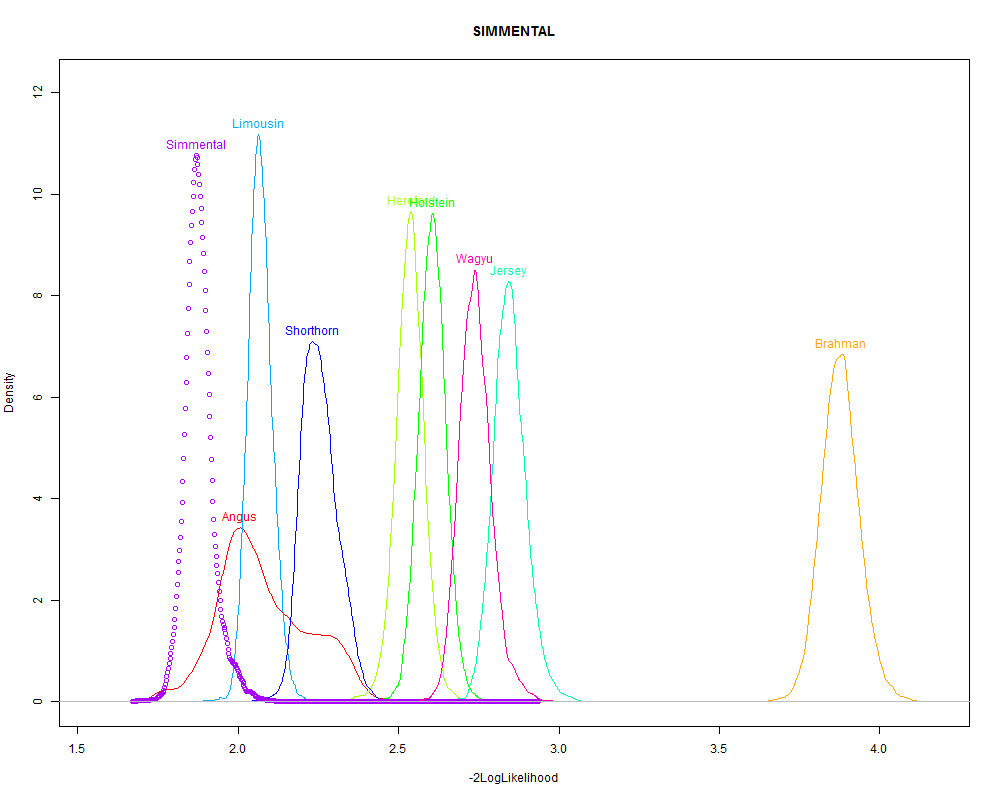


H


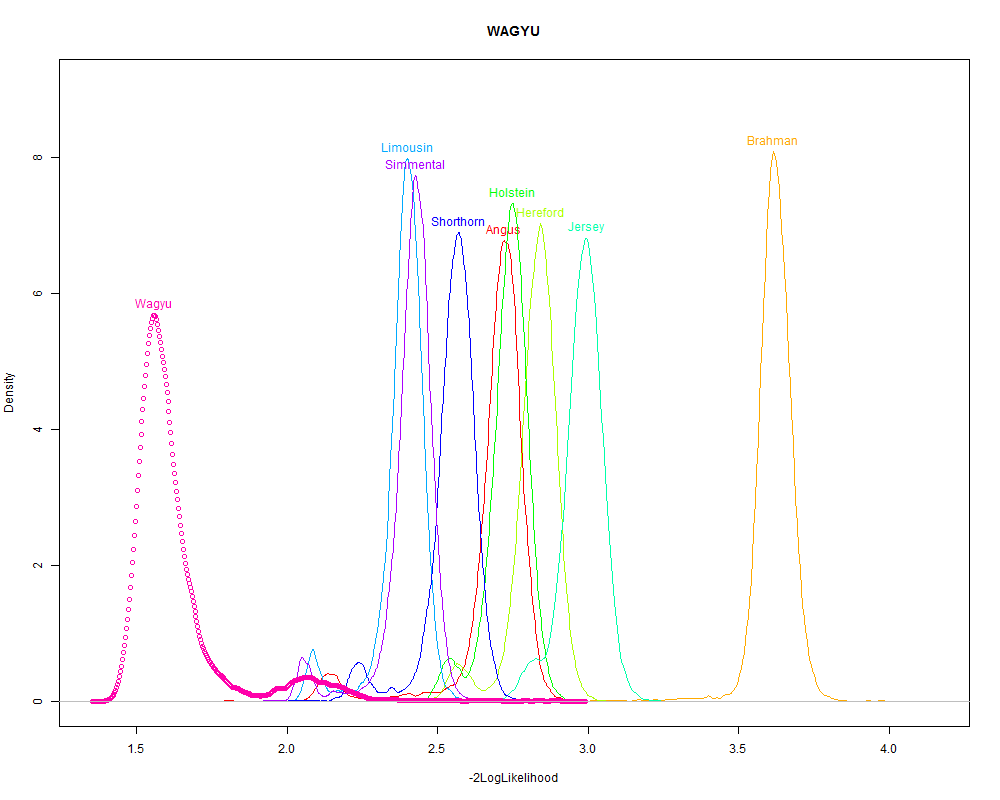


I
