# Supplementary material for: Estimation of Genomic Breed Composition for Purebred and Crossbred Animals Using Sparsely Regularized Admixture Models
Source: Front Genet. 2020 Jun 11;11:576. doi: 10.3389/fgene.2020.00576 (PMC7300184; doi:10.3389/fgene.2020.00576)
Supplement: Supplementary file 2 [file Data_Sheet_3.docx]

**Appendix**

**A Calculating a search direction to solve the GBC estimation**

The subgradient of $F\left( \mathcal{X} \right)$ in the model (4) with respect to a variable $\mathcal{X}_{j}$ is given by

$\partial_{j}F\left( \mathcal{X} \right)=\nabla_{j}L\left( \mathcal{X} \right)-\lambda sgn(\mathcal{X}_{j})$, (A1)

where $\partial_{j}F\left( \mathcal{X} \right)=\partial_{\mathcal{X}_{j}}F\left( \mathcal{X} \right)$，$\nabla_{j}L\left( \mathcal{X} \right)=\nabla_{\mathcal{X}_{j}}L\left( \mathcal{X} \right)$， and the set-valued function $sgn(\mathcal{X}_{j})$ (Bertsekas et al., 2003) is given by

$sgn(\mathcal{X}_{j})\triangleq\left\{ \begin{aligned} sign\left( \mathcal{X}_{j} \right), \mathcal{X}_{j}\neq0 \\ \left[ -1,1 \right], \mathcal{X}_{j}=0 \end{aligned}. \right.$ (A2)

Because of the subgradient of $F\left( \mathcal{X} \right)$ is separable in the variables, the problems of computing the minimum-norm element of the subgradient is also separable. Hence, we can solve the minimum-norm problem coordinate-wise to yield that the element of the minimum-norm subgradient with respect to a variable $\mathcal{X}_{j}$ is:

(A3)

where $\tilde{\nabla}_{j}F\left( \mathcal{X} \right)=\tilde{\nabla}_{\mathcal{X}_{j}}F\left( \mathcal{X} \right)$. In the last case of (A3), the element of the minimum-norm subgradient is zero, because we can set $sign\left( \mathcal{X}_{j} \right)$ to $-\nabla_{j}L\left( \mathcal{X} \right)/\lambda$ to achieve a norm of zero.

A search direction to solve the sparse neural networks is as follows

$\mathcal{h}\left[ \kappa\right]\triangleq-\mathcal{H}^{-1}\left[ \kappa\right]\tilde{\nabla}F\left( \mathcal{X}\left[ \kappa\right] \right)$,

In dealing with practical problems, quasi-Newton methods (Dennis and More, 1997) allow us to replace the Hessian matrix $\mathcal{H}[\kappa]$ by an approximation $\mathcal{B}[\kappa]$. So, the search direction is changed as follows

$\mathcal{h}\left[ \kappa\right]\triangleq-\mathcal{B}^{-1}\left[ \kappa\right]\tilde{\nabla}F\left( \mathcal{X}\left[ \kappa\right] \right)$ (A4)

leading to iterations of the form $\mathcal{X}\left[ \kappa+1 \right]\mathcal{\leftarrow X}\left[ \kappa\right]+\iota\mathcal{h}\left[ \kappa\right]$. $\iota$ is selected by a line search to satisfy the condition as follows

$$F\left( \mathcal{X}\left[ \kappa\right]+\iota\mathcal{h}\left[ \kappa\right] \right)<F\left( \mathcal{X}\left[ \kappa\right] \right)$$

where ${\mathcal{\{B}}^{-1}\left[ \kappa\right]\}$ is generated by

$\mathcal{B}^{-1}\left[ \kappa+1 \right]=\mathcal{B}^{-1}\left[ \kappa\right]+\frac{\mathcal{s[}\kappa\boldsymbol{]}\mathcal{s}^{\boldsymbol{'}}\boldsymbol{[}\kappa\boldsymbol{]}}{\mathcal{I}^{\boldsymbol{'}}\boldsymbol{[}\kappa\mathcal{]s[}\kappa\boldsymbol{]}}\left( \frac{\mathcal{I}^{'}\boldsymbol{[}\kappa\boldsymbol{]}\mathcal{B}^{-1}\left[ \kappa\right]\mathcal{I[}\kappa\boldsymbol{]}}{\mathcal{I}^{\boldsymbol{'}}\boldsymbol{[}\kappa\mathcal{]s[}\kappa\boldsymbol{]}}+1 \right)-\frac{\boldsymbol{1}}{\mathcal{I}^{\boldsymbol{'}}\boldsymbol{[}\kappa\mathcal{]s[}\kappa\boldsymbol{]}}\left( \mathcal{s[}\kappa\boldsymbol{]}\mathcal{I}^{\boldsymbol{'}}\boldsymbol{[}\kappa\boldsymbol{]}\mathcal{B}^{-1}\left[ \kappa\right]+\mathcal{B}^{-1}\left[ \kappa\right]\mathcal{I[}\kappa\boldsymbol{]}\mathcal{s}^{\boldsymbol{'}}\boldsymbol{[}\kappa\boldsymbol{]} \right)$ (A5)

with a scaled identity matrix approximation $\mathcal{B}^{-1}\left[ 0 \right]\triangleq\sigma\boldsymbol{I}$ for some positive $\sigma$, $\mathcal{I}\left[ \kappa\right]\boldsymbol{=}\tilde{\nabla} \tilde{F}\left( \mathcal{X}\left[ \kappa+1 \right] \right)-\tilde{\nabla} \tilde{F}\left( \mathcal{X}\left[ \kappa\right] \right),$ and $\mathcal{s}\left[ \kappa\right]\mathcal{=X}\left[ \kappa+1 \right]-\mathcal{X}\left[ \kappa\right]$. Typically, for large-scale problems, the approximation is constructed using limited-memory Broyden-Fletcher-Goldfarb-Shanno (L-BFGS) (Nocedal,1980). In L-BFGS, we do not explicitly store $\mathcal{B}^{-1}\left[ \kappa\right]$ but rather store a set of different $\mathcal{s}\left[ \kappa\right]$ and $\mathcal{I}\left[ \kappa\right]$.

**Active-set Method to Set Some Parameters to Zero**

To precisely set variables $\mathcal{X}\left[ \kappa\right]$ to zero, we use one of the active-set methods, which are widely used for solving L1-norm regulation problems (Krishnan et al., 2007). The variables are divided into two sets: the working set $\mathcal{W}$ containing the sufficiently non-zero variables, and the active set $\mathcal{A}$ containing the sufficiently zero-values variables.

The working set is defined as follows:

.

On each iteration, we take a projection of the Newton step along the working set using the Hessian matrix approximation $\mathcal{B}^{-1}\left[ \kappa\right]$ and a projected minimum norm subgradient for the active-set variables:

$\mathcal{X}_{\mathcal{W}}\left[ \kappa+1 \right]\leftarrow\mathcal{P}_{\mathcal{O}}\left( \mathcal{X}_{\mathcal{W}}\left[ \kappa\right]-\iota\mathcal{B}_{\mathcal{W}}^{-1}\left[ \kappa\right]\tilde{\nabla}_{\mathcal{W}}\tilde{F}\left( \mathcal{X}\left[ \kappa\right] \right) \right)$,

$\mathcal{X}_{\mathcal{A}}\left[ \kappa+1 \right]\leftarrow\mathcal{P}_{\mathcal{O}}\left( \mathcal{X}_{\mathcal{A}}\left[ \kappa\right]-\iota\mathcal{D}\left[ \kappa\right]\tilde{\nabla}_{\mathcal{A}} \tilde{F}\left( \mathcal{X}\left[ \kappa\right] \right) \right)$, (A7)

where we use $\tilde{\nabla}_{\mathcal{A}} F\left( \mathcal{X}\left[ \kappa\right] \right)$ to denote the sub-vector of $\tilde{\nabla} F\left( \mathcal{X}\left[ \kappa\right] \right)$ corresponding to elements of $\mathcal{A}$ and $\mathcal{B}_{\mathcal{W}}^{-1}\left[ \kappa\right]$ to denote the sub-matrix of $\mathcal{B}^{-1}\left[ \kappa\right]$ with all rows and columns of $\mathcal{W}$; $\mathcal{P}_{\mathcal{O}}$ is an orthant projection, which is effective at sparsifying the parameter vector and ensures that the line search does not cross points of non-differentiability, and is defined:

$\mathcal{P}_{\mathcal{O}}\left( \mathcal{X+h} \right)\boldsymbol{\triangleq}\left\{ \begin{aligned} 0 if \mathcal{X}_{j}\left( \mathcal{X}_{j}+\mathcal{h}_{j} \right)<0 \\ \mathcal{X}_{j}+\mathcal{h}_{j} otherwise \end{aligned} \right.$, (A8)

and $\mathcal{D}\left[ \kappa\right]$ is the diagonal scaling matrix and set to $\sigma^{-1}\left[ \kappa\right]\mathbf{I}$ with the Barzilai-Borwein scaling $\sigma^{-1}\left[ \kappa\right]\left( \sigma\left[ \kappa\right]\triangleq\frac{\mathcal{I}^{'}\boldsymbol{[}\kappa\mathcal{]I[}\kappa\boldsymbol{]}}{\mathcal{I}^{\boldsymbol{'}}\boldsymbol{[}\kappa\mathcal{]s[}\kappa\boldsymbol{]}} \right)$ (Fletcher,2001).

**B Algorithm**

We outline a general framework that can be used as a basis for implementing a Subgradient-based quasi-Newton method for solving the problem as follows:

Input: Function $L\left( \mathcal{X} \right)$, regulation parameters $\lambda$, initial random parameter $\mathcal{X}[0]$, optimality tolerate $\tau$, direction calculation function dir($\kappa,\boldsymbol{\eta},\sigma,\mathcal{S,T}$).

$\kappa\leftarrow0;\boldsymbol{\eta\leftarrow}\mathrm{NULL};\sigma=1;\mathcal{S\leftarrow}\mathrm{NULL}\mathcal{;T\leftarrow}\mathrm{NULL}\boldsymbol{;}$

1. $\tilde{F}\left( \mathcal{X}\left[ \kappa\right] \right)\leftarrow L\left( \mathcal{X}[0] \right)-\sum_{j=1}^{k} \lambda|\mathcal{X}_{j}[0]|$; //initial square error function, see (4).
2. $\boldsymbol{\eta}\left[ \kappa\right]\boldsymbol{\leftarrow}\tilde{\nabla}F\left( \mathcal{X}\left[ 0 \right] \right)$; // compute subgradient with the minimum norm, see (A3).
3. while $\left\| \boldsymbol{\eta}\left[ \kappa\right] \right\|_{\boldsymbol{inf}}\boldsymbol{>}\tau$
4. $\mathcal{h}\left[ \kappa\right]\leftarrow$dir($\kappa,\boldsymbol{\eta},\sigma,\mathcal{S,T}$);
5. $\mathcal{X}\left[ \kappa+1 \right]\leftarrow\mathcal{P}_{\mathcal{O}}(\mathcal{X}\left[ \kappa\right]+\iota\mathcal{h}\left[ \kappa\right])$; //compute new variable vector, see (A8).
6. $F\left( \mathcal{X}\left[ \kappa+1 \right] \right)\leftarrow L\left( \mathcal{X}[\kappa+1] \right)-\sum_{i=1}^{k} \lambda|\mathcal{X}_{i}[\kappa+1]|$; // compute new approximate square error function, see (4).
7. $\boldsymbol{\eta}\left[ \kappa+1 \right]\boldsymbol{\leftarrow}\tilde{\nabla}F\left( \mathcal{X}\left[ \kappa+1 \right] \right)$; // compute new subgradient with minimum norm(A3).
8. $\mathcal{s}\left[ \kappa\right]\mathcal{=X}\left[ \kappa+1 \right]-\mathcal{X}\left[ \kappa\right]$; $\mathcal{I}\left[ \kappa\right]\boldsymbol{=\eta}\left[ \kappa+1 \right]-\boldsymbol{\eta}\left[ \kappa\right]$;
9. $\mathcal{S\leftarrow[S s}\left[ \kappa\right]\boldsymbol{]}$; $\mathcal{T\leftarrow[T I}\left[ \kappa\right]\boldsymbol{]}$; $\sigma\leftarrow\frac{\mathcal{I}^{'}\boldsymbol{[}\kappa\mathcal{]I[}\kappa\boldsymbol{]}}{\mathcal{I}^{\boldsymbol{'}}\boldsymbol{[}\kappa\mathcal{]s[}\kappa\boldsymbol{]}}$ ; $\kappa\leftarrow\kappa+1$;
10. end while

Function dir() //Direction calculation in active-set method

Input: Iteration number $\kappa$, subgradient with minimum norm $\boldsymbol{\eta}$, $\sigma$, quasi-Newton matrices $\mathcal{S,}$ and $\mathcal{T}$.

Output**:** Descent direction $\mathcal{h}$

1. $\mathcal{h\leftarrow}\boldsymbol{0}$;$\mathcal{E\leftarrow}0.001$;;$\mathcal{A}\leftarrow\mathcal{W}^{c}$;
2. if $\kappa=0$
3. then $\mathcal{h\leftarrow}\boldsymbol{-}min\{1, \frac{1}{\left\| \boldsymbol{\eta} \right\|}\}\boldsymbol{\eta}$;
4. else $\mathcal{h}_{\mathcal{A}}\boldsymbol{\leftarrow-} \sigma\boldsymbol{\eta}_{\mathcal{A}}$, $\mathcal{h}_{\mathcal{W}}\boldsymbol{\leftarrow-} \mathcal{B}_{\mathcal{W}}^{-1}\boldsymbol{\eta}_{\mathcal{W}}$; // apply L-BFGS algorithm, see (A 7)
5. end if

**REFERENCES**

Dennis, J., and More, J. (1997). Quasi-newton methods, Q18 motivation and theory. SIAM Rev. Soc. Ind. Appl. Math. 19, 46–89. doi: 10.1137/1019005

Fletcher, R. (2001). On the Barzilai-borwein Method. Technical Report. University of Dundee.

Krishnan, D., Lin, P., and Yip, A. (2007). A primal-dual active-set method for nonnegativity constrained total variation deblurring problems. IEEE Trans. Image Process. 16, 2766–2777. doi: 10.1109/TIP.2007.908079

Nocedal, J. (1980). Updating quasi-newton matrices with limited storage. Math. Comput. 35, 773-782. doi: 10.1090/S0025-5718-1980-0572855-7
